# Proposed Strategies to Improve Adult Asthma Management in Egypt: Expert Review and Recommendations

**DOI:** 10.5334/aogh.3782

**Published:** 2022-11-17

**Authors:** Hossam Hosny, Ashraf Madkour, Mohamed Hantera, Mohamed Dahy, Faten Emara, Maha Ibrahim, Tarek Safwat

**Affiliations:** 1 Kasr Al-Aini, Faculty of Medicine, Cairo University, Egypt; 2 Faculty of Medicine, Ain Shams University, Egypt; 3 Faculty of Medicine, Tanta University, Egypt; 4 National Health Insurance Organization, Egypt; 5 Specialized Medical Centers Organization, Egypt

**Keywords:** Asthma, inflammatory, bronchitis, smoke, Egypt

## Abstract

**Background:**

Several challenges face asthma management in Egypt, including the high percentage of uncontrolled patients, inadequate compliance, and overuse of short-acting beta-agonists (SABAs) leading to increased asthma-related morbidity and mortality. In this regard, the recent Global Initiative for Asthma (GINA) recommendations included inhaled corticosteroids containing therapy for mild asthma. Local healthcare systems and healthcare professionals (HCPs) often experience practical challenges when implementing global guidelines.

**Objective:**

The present expert review aims to outline the development of local guidelines and health policies that integrate global advances in asthma management while addressing unmet needs and challenges in Egypt.

**Methods:**

A steering committee of health policymakers and respiratory experts from the principal healthcare sectors in Egypt came together in March 2021 to develop a consent and national guideline for local asthma management, codifying the current challenges and the required elements for adequate control. The recommendations were either evidence-based or consensus-based from the clinical expertise and perspectives of the committee.

**Results:**

The committee identified vital challenges facing all chronic airway diseases with initial focus on asthma management in Egypt in diagnosis, data collection, policymaking, patients’ awareness, and physicians’ attitudes. In general, the committee stated that globally adapted management protocols necessitate addressing from diverse perspectives through policymakers, HCPs, and patients. Accordingly, it is vital to provide relevant education for the patient and HCPs. The recommendations emphasize key elements concerning baseline assessment, diagnosis, treatment strategy with regular review of patient progress, and compliance toward the introduced reforms.

**Conclusions:**

Full integration of these recommendations into local practice allows physicians to sustain adequate management while reducing preventable exacerbations and unnecessary burdens. The proposed strategies outline efficient patient-centered management that approaches asthma as an inflammatory condition, encouraging health promotion and patients’ compliance.

## I. Introduction

Asthma burden is on rise, affecting more than 339 million patients worldwide, with a prevalence of 12.6% [1,2]. In 2019, asthma-related mortality exceeded 461,000 deaths, mostly in low- and middle-income countries (LMIC), where early diagnosis and adequate treatment remain challenging [3]. In Egypt, the prevalence is around 6.7% of the general population [4].

The definition of asthma as bronchial hyper-responsiveness with symptomatic wheezing proved inefficient, given the effects of smoking in adult patients and the overlap with chronic bronchitis [5]. Today, guidelines define *asthma* as a chronic inflammatory condition within the airways displaying chest tightness, expiratory wheeze, and breathlessness in adult and pediatric populations [6]. It is worth mentioning that some common symptoms of asthma exacerbations, namely dry cough and progressive dyspnea, are characteristic of COVID-19, which contribute further to the diagnostic challenge [7].

Asthma adherence to treatment as well as asthma control in the Middle East and North Africa (MENA), including Egypt, is unsatisfactory, with less than one-quarter of asthma patients having good adherence [8] and less than one-third of patients with asthma having controlled disease [9].

Ideally, the development of stepwise management with reliever and controller medications has lowered exacerbation risks [10]. However, asthmatic attacks persist, and optimal asthma control remains elusive, especially in primary care settings [10]. In 2020, the Global Initiative for Asthma (GINA) identified that no evidence supported the long-term use of short-acting beta-agonist (SABA) alone, and SABA is no longer recommended as monotherapy for asthmatic patients [6].

Likewise, the under-use of inhaled corticosteroids (ICS) increased the risk of exacerbations and related mortality [11]. In contrast, single-inhaler combinations of long-acting beta-agonists (LABA) with ICS taken as needed to potentially overcome conventional approaches’ limitations, with considerable evidence over the past decade and from the recent SYGMA trials [12,13].

In 2021, GINA recommended as-needed ICS/Formoterol, particularly Formoterol, for patients with mild symptoms and across all asthma steps, whereas SABA alone was no longer preferred [13]. This modification outlines a substantial shift in treatment strategy, prioritizing long-term safety and exacerbation reduction, even in mild patients [14].

To facilitate tangible improvements in disease control, these changes must be assimilated into routine clinical practice, particularly in primary care settings, where most patients are present [14]. Indeed, the emergence of local guidelines is usually inadequate to reflect global changes, especially in developing countries [10,15]. Although the adverse effects of SABA over-reliance became evident, entrenched beliefs and psychological reliance on conventional therapy persist [16,17].

The present article aims to outline the development of local guidelines and health policies that integrates global advances in chronic lung diseases, particularly asthma, management while addressing unmet needs and challenges in Egypt. The article also proposes integrated strategies to tackle healthcare infrastructure and policy challenges, early diagnosis and prevention, unified guidelines and protocols, patient compliance and sustainable care, and, eventually, best practices toward a call-to-action for asthma control.

## II. Materials and Methods

In March 2021, AstraZeneca organized a lung forum summit with a steering committee of health policymakers and respiratory experts from the principal healthcare sectors in Egypt to elaborate a proposed strategy for local asthma management, tackling current challenges in managing patients and reducing disease burden, and codifying the required elements for adequate care. The recommendations outlined in this article and obtained as outcomes of the summit were either evidence-based or consensus-based from the clinical expertise and perspectives of the committee.

During this meeting, a systematic review of the current practical aspects of the treatment algorithm for chronic lung diseases, particularly asthma, was done. A steering committee composed of key physicians from Egypt was responsible for discussing and providing consent on the burden and management of asthma patients in Egypt.

## III. Outcomes and Recommendations

### 1. Epidemiology and burden of chronic respiratory diseases (CRDs)

Frequent CRDs in Egypt include asthma, chronic obstructive pulmonary disease (COPD), and lung cancer, designating a growing burden on local communities and healthcare systems. This evolving threat restricts medical societies’ resilience to external challenges and confines their potential for recovery and sustainability. The BREATHE study estimated the prevalence of COPD in Egypt around 3.5% of the general population, whereas the rate of exacerbations reached 41.9% [18,19]. The gender-specific distribution was higher in males with a frequency of 6% [19].

Globally, asthma accounts for 1 in every 250 deaths [20]. Although overall mortality has declined since the 1980s, the global prevalence increased by 50% each decade [21]. In developing countries, asthma increases as communities become urbanized, which correlates with higher atopic sensitization and allergic conditions such as rhinitis [22].

The enriched awareness of asthma manifestations enhanced the diagnostic sensitivity, thus increasing the reported prevalence [23]. Likewise, the rapidly changing lifestyles in urbanizing regions potentially exposed a hidden population susceptible to developing the condition but not exposed to risk factors [24].

In Egypt, the SNAPSHOT study concluded that over 6.7% and 26.5% of the general adult and pediatric population, respectively, have asthma [4]. The highest prevalence was noted in Greater Cairo and the northern portion of the country, where most urban populations live [4]. Meanwhile, disparate definitions and tools in epidemiological studies have hindered accurate estimation of the prevalence rate, deviating from 34% over-diagnosis to 54% under-diagnosis [25]. Population surveys employed different questionnaires, and the diagnosis of asthma depended on the participants themselves [26]. However, a comparison between patients and physicians reported prevalence revealed half of the population as undiagnosed [27].

The incremental costs for asthma approach $4.3, $20, and $56 billion in Canada, the European Union, and the United States, respectively [28]. Asthma potentially leads to substantial limitations on patients’ physical, psychological, and professional lives [29]. This growing burden endangers healthcare systems’ resilience to novel challenges and capacity for recovery and sustainability [28]. Up to 22% of patients with mild symptoms encounter at least one exacerbation each year, granting the regular usage of SABA alone more safety risks and unfavorable outcomes [30]. Furthermore, low education levels and inadequate communication with health care professionals (HCPs) intensify this burden [31]. One study observed that only 20% of cases used their inhaler correctly at conventional times [32].

The situation is no different in Egypt. According to the ESMAA (Assessment of Asthma Control in Adult Asthma Population in the Middle East and North Africa) study, nearly half of the Egyptian patients are uncontrolled. Besides, nearly 75% of patients do not adequately adhere to proper asthma management plan [9].

### 2. Key challenges facing asthma management in Egypt (Figure 1)

**Figure 1:**
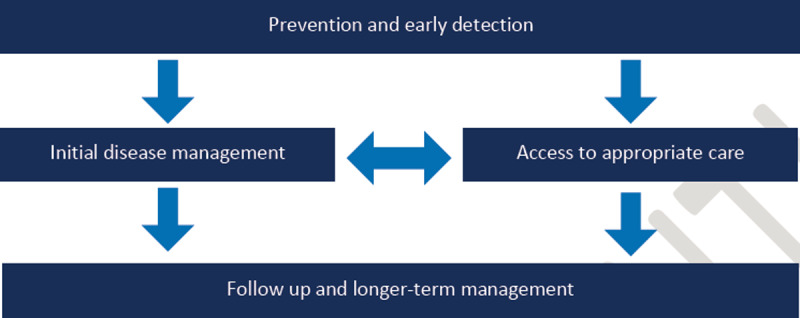
A key challenge facing asthma management in Egypt is the overlap in the routine care provided for patients with asthma.

#### 2.1. Policymaking challenges

Although global best-practice guidelines like GINA exist to improve clinical outcomes in different world regions, they have no routine implementation at the local level [33]. The shortage of political support, funding, or incentives possibly contributes to this disparity [33].

The committee identified several other factors that further exacerbate the challenge, including unaffordable medications, unclear plans for treatment dissemination, and lack of viable strategies for targeted communication and education.

#### 2.2. Challenges with physicians’ attitude

Physicians may lack the time or training needed, have insufficient up-to-date knowledge of recommendations, or resist the change [34,35]. Their conventional dependence on SABAs prescription and oversensitivity of corticosteroid side effects possibly restrain them from prescribing ICS [36].

The committee recognized another challenge facing primary care physicians in Egypt due to the lack of clear thresholds to trigger a referral to a specialized clinic. Likewise, their clinics usually lack the devices GINA recommends for asthma monitoring and evaluation.

#### 2.3. Diagnostic challenges

The overlap in the routine care provided for asthmatic patients and other respiratory conditions is a critical challenge facing asthma management in Egypt. Because the clinical symptoms for asthma are non-specific, it is usually misdiagnosed, particularly in the absence of objective tools such as spirometry [37]. Misdiagnosis delays appropriate therapy and escalate the risk of exacerbations and airway remodeling [27].

In Egypt, the lack of unified guidelines for the initial disease management depicts a challenging component within the public sectors of the Ministry of Health (MOH) and the National Health Insurance Organization (NHIO). Currently, there are two different guidelines for asthma management in MOH adopted from GINA, which hinders the potentials of a national registry because it requires a standard approach for diagnosis, treatment, and outcomes. Furthermore, the level of healthcare service provided is not standardized according to CRDs grade and severity.

The committee also pointed that sometimes asthma is mistakenly identified as a COPD exacerbation, which leads to misdiagnosis and mismanagement. Likewise, the late diagnosis of asthma reflects the frequent misconception of recurrent common cold or influenza attacks.

#### 2.4. Challenges in data collection

In this regard, current electronic medical records (EMRs) cannot distinguish patients in need of review, mainly due to gaps in data infrastructure, which potentially hampers the integration of the EMR across various healthcare sectors in Egypt. The committee recognized that the employed EMR systems significantly vary across public healthcare. For instance, the NHIO and the Specialized Medical Centers Organization (SMCO) harness two separate systems with divergent guidelines.

Given the vacancy of routine alerts and data flows, it is not feasible to identify patients with uncontrolled conditions, those at risk of SABA overreliance, or those seeing multiple HCPs several times. On the contrary, quite a few barriers restrain the functional implementation of a national registry. The main limitation arises from the lack of incentives and time capacity for treating physicians to fill out patients’ records, let alone the pivotal encounters with financial support for registry-related diagnostic tools, given the confined accessibility of spirometry in primary care, the shortage of trained personnel, and the need for continuous documentation and data entry.

#### 2.5. Challenges with patients’ awareness

A crucial shortcoming for successful treatment with a controller therapy lies in patients’ poor adherence to management protocol [12]. Indeed, epidemiological reports indicated that participants used inhalers only when symptoms occur and neglected regular treatment when having no necessary incentives [38,39]. In this regard, patients preferred reliever remedies, which further expand the overuse of SABAs and mask the underlying airway inflammation till the advanced stages [40]. Even more, in regions where community pharmacies sell SABAs with no formal prescription, this further hamper national attempts to mandate ICS/Formoterol [41].

In Egypt, the lack of patients’ awareness of treatment strategy carries a considerable hindrance to asthma control, particularly with their full accessibility to SABA without a prescription. They frequently over-rely on SABA until they encounter a resisting attack and reach for emergency care, further stretching the burden on healthcare resources. A recent study of 93,604 asthmatic patients with SABA overuse (more than 6.5 canisters per year) showed a significantly higher incidence of asthma-related urgent care (OR = 7.68) and the need for hospitalization (OR = 5.37) with three times higher costs [42]. Patients’ awareness and education are critical to achieving integrated management in Egypt, especially where most asthmatic patients cannot distinguish different inhalers and generally prefer simple dosage forms such as oral capsules or tablets rather than inhalers.

Meanwhile, the inadequate level of long-term follow-up emerges from patients’ poor awareness and low proportions of patients with medical insurance [9]. According to the results of the ESMAA study, around half of asthma patients in Egypt experience daytime symptoms and sleep-awakening attacks that require rescue treatment more than two days each week, 41.2% of them with limited activities and 68.6% with reduced lung function less than 80% of what was expected [9].

Even though 97.1% of asthma patients are currently treated, only 51% receive a fixed regimen of ICS+LABA, whether alone or adjuvant, and only 22.6% showed good adherence to medication [9]. Based on the GINA criteria for uncontrolled asthma, the committee regarded the current level of asthma control in Egypt as insufficient, requiring further addressing in proactive follow-up, healthcare access, and patient education.

## 3. Potentials of asthma management reforms in Egypt

GINA-based asthma management significantly improved health-related quality of life for most patients irrespective of their disease severity [43]. Two recent meta-analyses of 94 studies and 16 randomized trials, respectively, revealed that adherence to ICS determined its importance (OR = 1.74), fewer concerns (OR = 0.50), and lower exacerbations and hospitalization rates [44,45].

The evidence implies that reforms to healthcare systems have a better chance for success when coordinated at the national level, adopting early diagnosis and regular ICS-LABA, rigorously connecting with patients’ values and clinicians’ perspectives, and strictly setting legislation to minimize smoking exposure [46,47]. The Finnish national asthma program significantly reduced the annual costs by 72%, from $2,976 to $839 per patient, despite a three-fold rise in the numbers of treated patients [47].

In Egypt, a budget impact analysis from the NHIO estimated an inherent saving of three billion EGP after three years when prescribing ICS/LABA in mild asthma instead of SABA [48].

## 4. Proposed strategies for asthma management reforms in Egypt

In general, the committee stated that globally adapted management protocols necessitate addressing from diverse perspectives through policymakers, HCPs, and patients. Accordingly, it is vital to provide relevant education for the patient and HCPs. The recommendations emphasize key elements concerning EMRs, objective diagnosis, appropriate treatment with regular review of patient progress, and compliance toward the introduced reforms.

### 4.1. Recommendations for healthcare institutions

Local protocols should address asthma as a chronic inflammatory disorder, in the first place, with a secondary airway narrowing that demands more than a single medication to control and more than symptomatic relief for management. A framework for the management of asthma patients should include severity, adherence, or control classification. Also, a medical council to map the framework for asthma management, including diagnosis or treatment protocols, and classifications is recommended.

According to their symptoms, suspected cases of asthma should receive a confirmatory assessment with spirometry as early as possible. However, those presenting with a history of suggestive symptoms of asthma, such as intermittent wheeze, shortness of breath, cough, sleep apnea, and chest tightness, should undergo further investigation with prioritized attention. Thus, the treatment journey of asthma patients should start by classifying asthma severity.

Concerning adherence follow-up, the experts stated that the patients should be followed every one to three months to assess asthma patients regarding adherence, compliance, and response. It was highlighted that the first follow-up visit should occur after three months from patient diagnosis and treatment initiation to avoid clinic overload, primarily if clinical improvement can only be assessed after three months. Nonetheless, the three-month interval was debated by some experts. A follow-up period after one month of diagnosis/treatment initiation was recommended according to GINA guidelines. If a compliant patient does not improve after one month and has no comorbidities, stepping up treatment or other treatment strategies shall be considered. Waiting for three months might lead to further complications.

Healthcare institutions need to update their reimbursement guidelines reflecting the up-to-date economic evaluation, cost of asthma care, and the value of current ICS regimens. They should also facilitate access to specialized care and specialized lung clinics enhanced with necessary objective diagnostic measures. Partnerships with manufacturers and pharmaceutical companies are essential to provide asthma clinics with required devices and facilities based on evidence-based decisions.

### 4.2. Recommendations for healthcare records

The committee emphasized the pivotal need for a national asthma registry to subdue the current disease burden and advocated for a unified EMR system where physicians’ evaluations correspond to their records filing rate.

The EMR process must be integrated and harmonized within NHIO, MOH, and Universal Health Insurance Authority (UHIA) by adopting the same recording system, whether Oracle or SAP.

The prospective registry should establish partnerships among all healthcare service providers while relying on best practices from successfully established registries in Egypt, such as the National Cancer and Hepatitis Registries.

The registry should encompass a temporal framework incorporating patients’ history and journey from early stage, with a flowchart whenever they visit the hospital or the outpatient clinic covering their signs, symptoms, interventions, consultations, treatments, and complications. Records should also set average values for spirometry results based on local data distribution.

Adopting a process, resources, and outcomes systems for informatics infrastructure, the committee advised developing integrative IT resources with robust process and friendly interface to all stakeholders and reliable key performance indicators (KPIs).

An established three-year plan should tackle the resource gaps by training HCPs personnel to follow specified filing systems, digitizing the current paper system to extract data properly, allocating different forms of funds, and eventually formulating a core team for implementation and linkage among stakeholders.

### 4.3. Recommendations for HCPs

Primary care physicians should effectively manage asthma in the primary care setting and refer complicated cases to a specialized lung clinic with further expertise. They should be competent with the benefits and adverse events of tailored medications, the clues to upgrade the regimen or seek a specialist consultation, and the management strategies following exacerbations and during maintenance therapy.

Physicians should receive proper training to comprehend the concept of asthma control and reversible versus irreversible airway obstruction while acquiring skills to appraise contemporary literature. They should receive continuous involvement in the protocols’ evolutionary process and evaluation to guarantee their adherence and cooperation.

A local medical council should provide relevant training and education for HCPs encouraging appropriate early ICS use, disregarding SABA monotherapy and annual prescription of at least three canisters and ensuring a better understanding of asthma pathogenesis and up-to-date literature.

Treating physicians should readjust the therapeutic regimen in the following week and review their management protocol each one to three months, whichever is feasible, to evaluate patients’ adherence, satisfaction, compliance, and response and measure lung functions with either peak flow rate or spirometry. Also, they should devise a detailed history addressing environmental triggers and occupational exposures.

The follow-up review must address possible comorbidities, including rhinosinusitis, bronchiectasis, obstructive sleep apnea, and gastric dysfunction. If a compliant patient does not improve after the proposed time frame and has no evidence for comorbidities, stepping up or shifting treatment plans shall be considered.

### 4.4. Recommendations for patients’ compliance

Differentiation between awareness and educational activities is critical because both need to be addressed with the relevant tools. Awareness campaigns deliver a general understanding of the condition, possible treatment, and responsibilities. At the same time, educational events teach patients how to use their inhalers once prescribed.

Because 29% of Egypt’s population is still illiterate [49], the committee recommended the national distribution of educational videos instructing patients to use their inhaler devices correctly while training nurses to educate the mothers of affected children. Also, the value of physicians’ direct education is evident in improving patients’ satisfaction and daily function and even reducing hospitalizations [50,51].

Global awareness events such as World Asthma Day can be utilized to further disseminate awareness among patients. Campaigns should address patients’ fear, stigma, misconceptions, and inability to accept the inhaler.

After awareness and education, there should be compliance. Patients should understand the difference between SABA and ICS therapy and the disadvantages associated with SABA overuse. They should learn to monitor their asthma at home, measuring the frequency and degree of symptoms and possibly estimating their peak expiratory flow rate. Eventually, further legislation should prohibit SABA purchases without formal prescriptions.

## 5. Conclusion

Asthma management in Egypt faces several challenges due to shortage of funding, insufficient up-to-date knowledge among treating physicians, delayed diagnosis, lack of unified guidelines, and limited patient awareness. In this report, the experts from Egypt highlighted several recommendations to meet these needs. The experts also highlighted the overuse of SABAs in asthma management, leading to multiple hazards in mortality and morbidity. There is a need to raise the knowledge about recent GINA recommendations concerning the use of ICS plus a rapid-onset bronchodilator as the preferred as-needed inhaler across all steps of asthma care. The recommendations of the present article potentially translate these global guidelines into local practice entailing collaborative effort from physicians, healthcare professionals, and policymakers. The proposed strategies outline efficient patient-centered management that approaches asthma as an inflammatory condition encouraging health promotion and patients’ compliance and adherence to treatment. Ideally, establishing a national asthma registry with unified local guidelines, educated patients, and trained medical personnel underscores better possibilities to minimize asthmatic exacerbations and their associated burdens on healthcare while improving the overall disease control and patients’ quality of life.

## Figures and Tables

**Table:** 

SABAs	short-acting beta-agonists
GINA	Global Initiative for Asthma
HCPs	healthcare professionals
LMIC	Low- and middle-income countries
MENA	Middle East and North Africa
ICS	inhaled corticosteroids
LABA	long-acting beta-agonists
CRDs	chronic respiratory diseases
COPD	chronic obstructive pulmonary disease
ESMAA	Assessment of Asthma Control in Adult Asthma Population in the Middle East and North Africa
MoH	Ministry of Health
NHIO	National Health Insurance Organization
EMRs	electronic medical records
SMCO	Specialized Medical Centers Organization
UHIA	Universal Health Insurance Authority
KPIs	key performance indicators
